# The roles and clinical applications of interleukins in endometrial carcinoma

**DOI:** 10.3389/fonc.2022.1001693

**Published:** 2022-11-30

**Authors:** Yuqin Zang, Huanrong Li, Shiqi Liu, Ruqian Zhao, Kaiwen Zhang, Yuqi Zang, Yingmei Wang, Fengxia Xue

**Affiliations:** ^1^ Department of Gynecology and Obstetrics, Tianjin Medical University General Hospital, Tianjin, China; ^2^ Tianjin Key Laboratory of Female Reproductive Health and Eugenics, Tianjin Medical University General Hospital, Tianjin, China; ^3^ Hangzhou College of Preschool Teacher Education, Zhejiang Normal University, Hangzhou, China

**Keywords:** interleukin, endometrial cancer, expression, mechanism, clinical application

## Abstract

As a common malignant tumor of the female reproductive system, endometrial carcinoma (EC) seriously endangers women’s health with an increasing incidence. The oncogenesis and progression of cancer are closely linked with immune microenvironment, of which interleukins are the important components. In order to illustrate the roles and clinical applications of interleukins in EC, literature of interleukins and EC were reviewed. Based on the present studies, interleukins play crucial roles in the oncogenesis and development of EC *via* regulating the proliferation, migration, invasion, angiogenesis, apoptosis, pyroptosis and autophagy of EC as well as the immune function against EC. And some of the interleukins seems to have prospective clinical applications in EC, such as evaluating the risk of tumorigenesis, discriminating the malignancy from benign disorders or normal condition, indicating cancer aggressiveness, predicting the prognosis of patients and serving as the novel therapy. However, there is still a long way to go before the clinical applications of interleukins in EC come into reality. Nevertheless, it is certain that the exploration of interleukins will definitely be of great benefit to the screening, diagnosis and treatment of EC in the future.

## Introduction

As a common malignant tumor of the female reproductive system, endometrial carcinoma (EC) seriously endangers women’s health (1, 2). As per the Global Cancer Statistics 2020 (3), there are more than 400,000 new cases of cancer at corpus uteri and caused nearly 100,000 deaths, the vast majority of which is EC. Based on the dependence on estrogen, EC is divided into two histological subtypes: the estrogen-dependent type I EC, which is also called endometrial adenocarcinoma (EAC), and estrogen-independent type II EC, such as the uterine serous papillary adenocarcinoma (USPC), clear cell carcinoma and undifferentiated carcinoma, which show more aggressiveness and poor prognosis when compared with type I EC (1, 4). And based on the genomic features proposed by The Cancer Genome Atlas (TCGA) Research Network in 2013, EC can be classified into four categories: POLE ultramutated, microsatellite instability hypermutated, copy-number low, and copy-number high (5). This kind of molecular classification can provide more reliable information for prediction of outcome and guidance for post-surgical adjuvant treatment of the patients. Due to the unhealthy lifestyle and prolonged lifespan, the incidence of EC has been increasing, which even makes EC the most common gynecological cancer particularly in development countries and causes a lot of death. Therefore, novel diagnostic biomarkers and therapeutic targets are needed for better management of EC.

**Figure 1:**
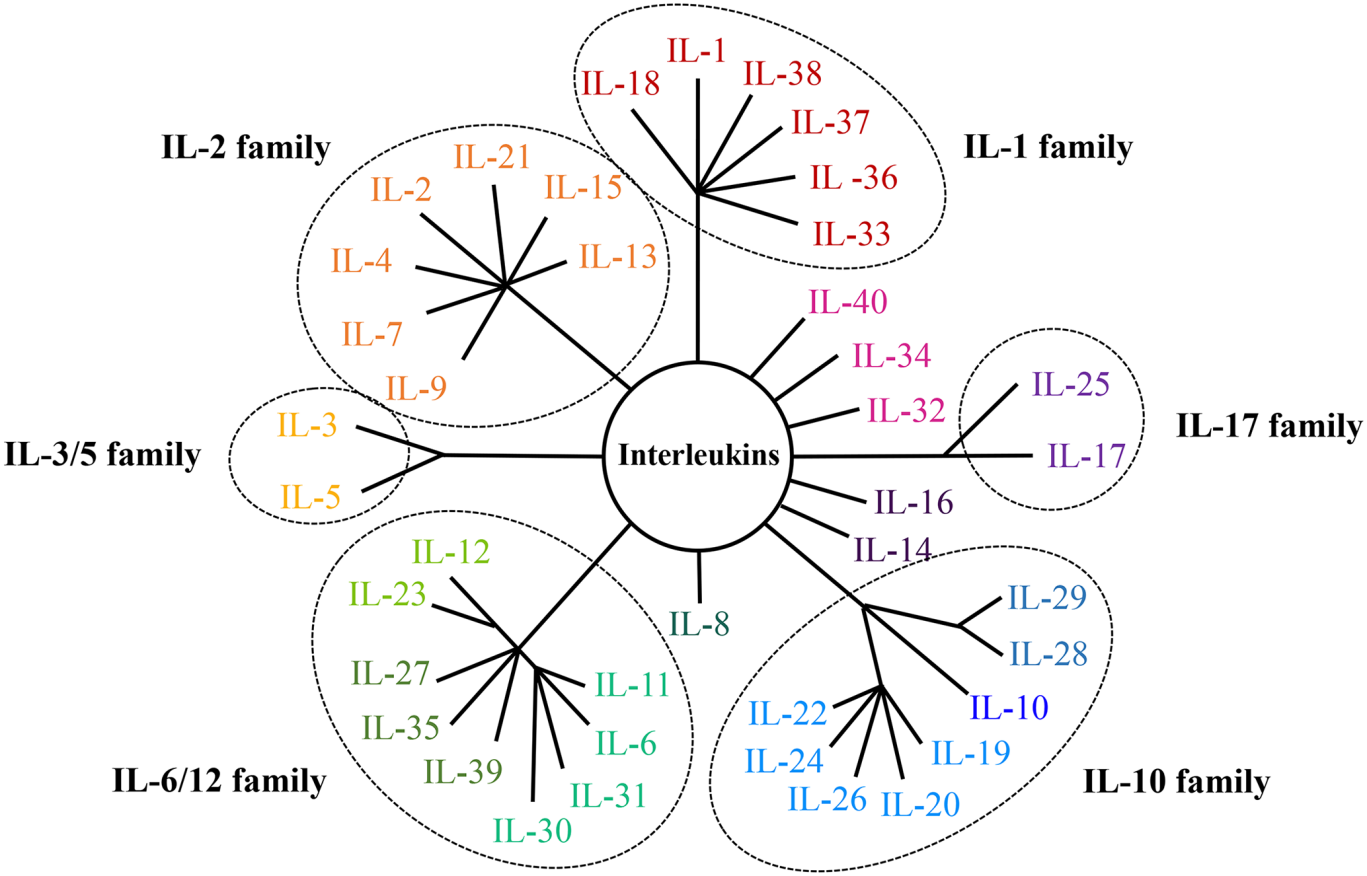
Classifications and structures of the interleukin family.

The oncogenesis and progression of cancer are closely linked with immune microenvironment, of which interleukins (ILs) are the important components. Interleukins are cytokines mainly synthesized by immunocytes and endothelial cells, and exhibit pleiotropic functions including modulating transcription factors, regulating inflammation and facilitating cell communication (6). There are dozens of interleukins which are categorized into several families, including the IL-1 family, IL-2 family, IL-3/5 family, IL-6/12 family, IL-10 family and IL-17 family (7–20). In addition, there are also some interleukins belonging to none of those families, like IL-8, IL-14, IL-16, IL-32, IL-34, IL-40 and other newly-discovered interleukins. Classifications and structures of interleukin families were displayed in **Figure 1**. According to the literature, interleukins play important part in the occurrence and development of cancer and may be used as diagnostic biomarkers and therapeutic targets. In order to illustrate the roles and clinical applications of interleukins in EC, studies of interleukins and EC were reviewed in this paper.

## Expression of interleukins in EC

### The IL-1 family

The IL-1 cytokine family is comprised of seven pro-inflammatory ligands (IL-1α, IL-1β, IL-18, IL-33, IL-36α, IL-36β and IL-36γ), four anti-inflammatory ligands including IL-1 receptor antagonist (IL-1RA), IL-36 receptor antagonist, IL-37 and IL-38 (8, 9). The expression levels of IL-1 family in EC were presented in **Table 1**.

**Table 1:** The expression of IL-1 family and IL-2 family in EC.

Authors	Year	Samples	Size	Method	ILs	Findings
Yang (21)	2020	Tissue	EC: 15, AH: 3 BGD: 6	IHC	IL-1β	IL-1β was overexpressed in EC and AH than in BGD.
Singer (22)	2002	Tissue	EC: 27, NED: 13	PCR ELISA	IL-1α IL-1β	1. IL-1α preferentially expressed in EC with moderate and poor differentiation than in normal endometrium and EC with well differentiation. 2. The mRNA of IL-1β was detected in most of the EC lesions but not normal endometrium.
Chopra (23)	1997	Serum	EC: 59, NC: 20	ELISA	IL-1α IL-1β IL-2 IL-7	1. Levels of IL-1α and IL-1β were not elevated in EC patients. 2. Levels of IL-2 and IL-7 were significantly elevated in patients with EC.
Van (24)	1991	Tissue	EC: 5, BGD: 5	PCR	IL-1α IL-1β	no difference was found between cancer and normal tissues.
Yron (25)	1986	Serum	EC:20, HC:27	ELISA	IL-2	Levels of IL-2 were lower in EC patients and slightly elevated after removing the tumor mass.
Wang (26)	2021	Tissue	EC:66	RNA-sequence	IL-32	IL-32 was overexpressed in EC and associated with longer overall survival time.
Zeng (27)	2020	Tissue	EAC: 260, BGD: 150	IHC ELISA	IL-33	IL-33 expression was elevated in EAC patients and increased with the differentiation grade.
Lan (28)	2020	Serum	EC: 42, BGD: 41 HC: 43	ELISA	IL-33	Levels of IL-33 were elevated in EC group.
Zeng (29)	2016	Serum	EAC: 160, HC: 160	ELISA	IL-33	Levels of IL-33 in EAC patients were elevated and related to adverse clinical characteristics.
Wang (30)	2021	Tissue	EAC: 56, NED: 62	IHC	IL-37	IL-37 expressions were decreased in EAC and related to adverse clinical characteristics.

ILs, interleukins; EC, endometrail cancer; AH, atypical hyperplasia; BGD, benign gynecologic disorder; NED, non-endometrial disease; NC, non-cancer; HC, healthy control; EAC, endometrial adenocarcinoma; IHC, immunohistochemistry; PCR, polymerase chain reaction; ELISA, enzyme-linked immunosorbent assay.

The IL-1 system is formed by two ligands IL-1α and IL-1β, two types of IL-1R, and the co-receptor IL-1R accessory protein, as well as the inhibitor IL-1RA (8, 22). The IL-1 system appears to have varied functions under different physiological or pathological conditions. As for the expression of IL-1α and IL-1β in EC, there remains controversy. For the expression in serum, no increase of IL-1α or IL-1β was reported in EC patients (23). For the expression in tissue, one study (24) claimed no difference between normal and cancer tissues, but two later studies (21, 22) covered an elevation in EC than in benign disorders. This difference may be caused by the limit on the sensitivity of detection in the earlier studies (23, 24) which were both done in the 1990s. Besides, it was declared that the expression of IL-1α in EC was independent of ovarian steroid receptor (22, 31), and reduced by drugs including toremifene (32), genistin (33), daidzin (33), Glycyrrhizae radix (34), Glycyrrhizin (34) and Chinese traditional medicine (35, 36) Juzen-taiho-to and Shimotsu-to. And IL-1β can be upregulated by leptin (37) and 2,3,7,8-tetrachlorodibenzo-p-dioxin (38), but downregulated by metformin (39) and weight loss (40).

IL-33 is another member of the IL-1 family, which joins in T helper 2 (Th2) cell immunity and plays an important role in the release of proinflammatory factors *via* the receptor ST2. The levels of IL-33 in serum and tissue were both significantly elevated in EC patients (27–29). In contrast with IL-1 and IL-33, the anti-inflammatory and anti-tumor cytokine IL-37 was downregulated in EC (30). As for the expression levels of IL-18, IL-36 or IL-38, there has been no report in EC. Nevertheless, it is worth noting that the serum level of IL-18 declined in patients with thickened endometrium after tamoxifen therapy for breast cancer, which is a risk for the tumorigenesis of EC, indicating that IL-18 may be downregulated in tamoxifen-derived EC (41).

### The IL-2 family

IL-2 was first discovered as a T-cell growth factor and signals *via* IL-2 receptor (IL-2R) which contains three subunits: IL-2Rα, IL-2Rβ and IL-2Rγ. IL-2Rα specifically binds to IL-2; IL-2Rβ binds not only to IL-2 but also to IL-15; and IL-2Rγ is shared by all members of IL-2 family including IL-2, IL-4, IL-7, IL-9, IL-13, IL-15 and IL-21 (10, 11). Studies of IL-2 family expression in EC were shown in **Table 1**.

As a multifunctional cytokine, IL-2 shows suppressing or promoting effects on tumor *via* regulating the propagation and function of natural killer (NK) cells or T cells (11). Reported by Yron et al. (25), the serum levels of IL-2 were lower in EC patients (stage I, n = 20) than that in healthy controls, and which were slightly elevated after removing the tumor mass. And they owed the reason of IL-2 deficiency in EC to the low amount of T cells and the existence of suppressor macrophages. However, reported by Chopra et al. (23), the serum levels of IL-2 were higher in EC patients (stage I, n = 20; stage II, n =8; stage III, n = 5; stage IV, n = 6) than that in women without cancer. The reason why the two results conflict is that the later study enrolled patients with advanced EC, which contributed to the higher level of IL-2 in EC group. Besides, the expression of IL-2 can be reduced by treatment with metformin (39) and intervention of weight loss (40).

IL-4 and IL-13 present similar functions for their neighboring encoding genes and analogous transcriptional regulatory elements (42). According to the reports, IL-4 was downregulated in EC patients after treated with megestrol acetate alone or combined with radix astragali (43), while IL-13 was not detectable in the serum of either EC or control group (44). IL-7 plays a crucial role in the survival and differentiation of lymphocyte *via* IL-7 receptor. The elevated serum levels of IL-7 were detected in EC patients (23), which decreased after losing weight (45). IL-21, signaling through the heterodimeric receptor composed of the common γ-chain of IL-2 family and the IL-21 receptor α-chain, was suppressed in Ishikawa cells dealing with chemotherapeutic drugs (46). The expression levels of IL-9 and IL-15 in EC have not been reported yet. But the IL-9^+^ lymphocyte infiltration was found to be related with the differentiation of tumor, survival of patients and expression of progesterone receptor in EC, indicating an association between IL-9 and EC (47).

### The IL-6/12 family

The members of IL-6/IL-12 family share a four-α-helix-bundle motif and belong to type 1 family of hematopoietic cytokines. Based on the structural feature and receptor type, IL-6, IL-11 and IL-31 are members of IL-6 subfamily (12); IL-12 and IL-23 are designated to IL-12 subfamily (7, 12); IL-27, IL-35 and IL-39 belong to both IL-6 and IL-12 subfamily (19, 20); and IL-30, which is originally identified as a subunit of IL-27, while exerts biological activities independently and shares homology with IL-6 subfamily members (7). **Table 2** presents the studies of IL-6/12 family expression levels in EC.

**Table 2:** The expression of IL-6/12 family in EC.

Authors	Year	Samples	Size	Method	ILs	Findings
Chen (48)	2022	Tissue	EC: 552, N: 23	RNA-sequence	IL-6	IL-6 was one of the downregulated genes in EC.
Lu (49)	2021	Tissue	EC: 25, BGD: 25	PCR	IL-6	Levels of IL-6 were higher in EC.
Shao (50)	2016	Serum	EC: 128, NED: 294	ELISA	IL-6	IL-6 levels were higher in EC patients and positively associated with EC risk.
Che (51)	2014	Tissue	EC: 86, NED: 50	IHC	IL-6	IL-6 levels were higher in EC.
Friedenreich (52)	2013	Serum	EC: 519, HC: 964	ELISA	IL-6	Levels of IL-6 were higher in EC patients.
Dossus (53)	2013	Serum	EC: 233, HC: 446	ELISA	IL-6	Levels of IL-6 were higher in EC cases.
Brooks (44)	2012	Serum	EC: 12, NC: 10	ELISA	IL-6 IL-12	1. No significant difference of IL-6 levels were found. 2. IL-12p70 levels were significantly reduced in EC patients.
Slater (54)	2006	Tissue	EAC: 20, NED: 10	IHC	IL-6	IL-6 was increased 4.4-fold in EAC than in normal endometrium.
Bellone (55)	2005	Tissue	EAC: 14, USPC: 10 NED: 3	PCR	IL-6	IL-6 levels were higher in patients with USPC when compared to patients with EAC or NED.
Bellone (55)	2005	Serum	EAC: 19, USPC: 13 HC: 20	ELISA	IL-6	1. IL-6 values in patients with EAC and USPC patients were higher than those in controls. 2. IL-6 values in USPC were 6.1-fold higher than that in EAC.
Chopra (23)	1997	Serum	EC: 59, HC: 20	ELISA	IL-6	IL-6 levels were not elevated in EC patients.
Ferdeghini (56)	1994	Serum	EC: 37	ELISA	IL-6	IL-6 was preferred to express in advanced EC.
Sales (57)	2010	Tissue	EAC: 30, NED: 10	PCR	IL-11	IL-11 was upregulated in EAC lesions.
Yap (58)	2010	Lavage Tissue	EC: 16, NED: 14	ELISA IHC	IL-11	IL-11 is upregulated in uterine lavage and endometrial cells in women with EC.
Zhou (20)	2018	Tissue	EC: 45, HC: 15	IHC	IL-27	IL-27 was downregulated in EC and gradually reduced with the decrease of differentiation degree.
Zeng (27)	2020	Tissue	EAC: 260, BGD: 150	IHC ELISA	IL-31	IL-31 expression was elevated in EAC patients and increased with the differentiation grade.
Zeng (29)	2016	Serum	EAC: 160, HC: 160	ELISA	IL-31	The levels of IL-31 in EAC patients were elevated and related to adverse clinical characteristics.

NC, non-cancer; USPC, uterine serous papillary adenocarcinoma.

IL-6, the core member of IL-6 cytokines family, was first discovered in 1973 as a soluble factor for stimulating B cells which was secreted by T cells (59). The signaling of IL-6 is transduced *via* a hexameric high-affinity complex composed of IL-6, IL-6 receptor α (IL-6Rα) and glycoprotein 130 (gp130, a common chain shared by the receptors of IL-6 subfamily) (60). Numerous evidence has supported that IL-6 linked chronic inflammation to cancer. As for the study of IL-6 in EC, it was significantly elevated in EC than in normal controls in most studies (49–54), except for two studies (23, 44) declaring no elevation and one study (48) reporting a downregulation. The discrepancy in these results may be ascribed to the different sensitivity of detection kit and tumor microenvironment. Furthermore, the levels of IL-6 in EC patients vary in different conditions. Lower serum levels of IL-6 were detected in EC patients after weight loss (45), patients receiving robotic hysterectomy than abdominal hysterectomy (61), and patients with slight diarrhea than severe diarrhea after pelvic chemoradiotherapy (62). Also, the expressions of IL-6 in EC cells are regulated by many factors, among which estrogen is the most potent one, which upregulated IL-6 by NF-κB pathway in an estrogen receptor (ER)-dependent way (51) and by G protein-coupled receptor 30-mediated ERK/MAPK pathway in an ER-independent way (63). In addition to the direct effect, estradiol can increase the secretion of IL-6 in mononuclear cells isolated from EC patients (64). Moreover, the expression of IL-6 of EC cell can be enhanced by ERK/NF-κB signaling (65), Yes-associated protein (YAP) (66), period circadian regulator 1 (67), ulipristal acetate (68), dipeptidyl peptidase IV (69) and co-culturation with human mesenchymal stem cells (70), while be reduced by treatment with progesterone (71), metformin (72), tranexamic acid (73), fucoxanthin (74) and curcumin (75). Besides, EC-associated fibroblasts secreted more IL-6 than normal fibroblasts did (60).

IL-11 is traditionally regarded as an anti-inflammatory cytokine, which signals *via* the IL-11 receptor α (IL-11Rα) and gp130 receptor complex. But its proinflammatory role in multiple inflammation-associated cancers has also been discovered in recent years. Sales et al. (57) highlighted that the signaling between prostaglandin F2α and F-prostanoid receptor positively regulated IL-11 expression by calcium-calcineurin-NFAT pathway, while RCAN1-4 could exert a opposite effect, suggesting that IL-11 expression in EC might be induced in response to inflammatory stimulation. In human EC tissue, IL-11 are not merely expressed in epithelial tumor cells but also in tumor-associated vascular cells and stroma as well as the infiltrating leukocytes (58). Both the levels of IL-11 mRNA and protein were dramatically elevated in EC tissues (57, 58). And the levels of IL-11 in uterine flushing of women with EC were also higher (58).

IL-12, the key member of IL-12 subfamily, was originally recognized as a NK cell-stimulatory factor in 1989. The IL-12 signal transduction occurred *via* a heterodimer consisting of IL-12 receptor β1 and IL-12 receptor β2 (12). IL-12 is a potent inducer of anti-tumor immunity and suppresses tumor development by facilitating leukocyte recruitment, enhancing cytotoxic responses and inhibiting angiogenesis (76). The circulating levels of IL-12 were considerably lower in EC patients (44), and elevated when treated with rBBX-01 (77). Besides, the expression level of IL-12 decreased in macrophages treated with EC cells when compared with untreated group (78), while the peripheral blood mononuclear cells (PBMCs) isolated from EC patients secreted more IL-12 and less IL-10 than that from healthy controls.

IL-27 is a member of both the IL-6 and IL-12 subfamily cytokines, whose action is mediated through a receptor composed of IL-27 receptor α (also known as WSX-1) and gp130 (20). IL-27 potentiates anti-tumor immunity by both central immnunomodulatory effect of stimulating the development of NK cells and cell toxic lymphocytes (CTLs) and local antitumor effect of exerting potent anti-angiogenic and anti-metastatic activities (12). As reported (20), the level of IL-27 in EC tissues was lower than that in normal endometrium, as well as that in KLE cells than Ishikawa and RL95-2 cells, and rapamycin administration brought an overexpression of IL-27 in EC cells.

IL-31 is the only exception to the ‘gp130 rule’ of IL-6 subfamily for its receptor consisting of IL-31 receptor α and oncostatin receptor β, through which IL-31 joins the immune responses and tumor progression (27, 29). As described in studies, the levels of IL-31 in serum and tissue were both elevated in EC patients compared to those in healthy controls (29) or patients with benign gynecological diseases (27).

Taken together, IL-6/12 family is the most widely studied interleukin family. Among them, IL-6, IL-11, and IL-31 were all overexpressed in EC, while IL-12 and IL-27 were both downregulated in EC. Besides, the rest members of this family, IL-23, IL-30, IL-35 and IL-39, have not been studied in EC yet.

### The IL-10 family

The IL-10 family contains the founding member IL-10, the IL-20 subfamily (IL-19, IL-20, IL-22, IL-24, IL-26) and the distant type III IFN-γ subfamily (IL-28A, IL-28B and IL-29) (13–15). All these members consist of six α-helix and connecting loops and bind to class II cytokine receptors. The receptors are composed of specific receptor chains and common receptor chains IL-10 receptor 2 (IL-10R2) or IL-20 receptor 2 which are shared by the family (15). For example, the receptor of IL-10 contains two molecules of IL-10R1 and two molecules of IL-10R2 (13).

IL-10 plays a paradoxical role in immunity as well as in cancer (13–15). On the one hand, IL-10 inhibits the inflammatory Th17 cells and macrophages and thereby facilitates tumor onset and progression. On the other hand, IL-10 promotes the proliferation of CD8^+^ T cells and its cytotoxicity to tumor cells. As shown in **Table 3**, one study (44) detected no significant difference of IL-10 level between EC and normal controls, but two studies (23, 81) showed that the serum and tissue IL-10 concentrations were significantly higher in EC patients. In addition, the expression of IL-10 in immune cells have been investigated too. It was observed that IL−10 secreted by PBMCs after lipopolysaccharide stimulation was diminished in patients with EC than controls (44), while IL-10 secretion by Treg cells from patients with EC and healthy controls was not significantly different (84). What’s more, the level of IL-10 in EC-associated U937 cells was higher than that in untreated U937 cells (78). And IL-10 expression by THP-1 cells was increased when treated with exosomes isolated from hypoxic EC cells compared with normoxic EC cells (85). The discrepancy of these results might be due to the difference of tumor microenvironment which modulates the expression and function of IL-10. At the beginning of tumor genesis, IL-10 might predominantly act as a antitumor factor by potentiating the cytotoxicity of NK cells and CTLs to tumor cells; while with the progression of tumor, IL-10 might mainly act as a potent tumor promoter *via* the IL-10R expressed on tumor cells (14).

**Table 3:** The expression of the other interleukins in EC.

Authors	Year	Samples	Size	Method	ILs	Findings
Lu (49)	2021	Tissue	EC: 25, BGD: 25	PCR	IL-8, IL-17	Levels of IL-8 and IL-17 were higher in EC.
Kotowicz (79)	2017	Serum	EC: 118, HC: 49	ELISA	IL-8	IL-8 was overexpressed in EC patients and was an independent prognostic factor for EAC patients.
Ciortea (80)	2014	Plasma	EC: 44, NED: 44	ELISA	IL-8	Levels of IL-8 were elevated in EC group.
Chopra (23)	1997	Serum	EC: 59, HC: 20	ELISA	IL-8, IL-10	Levels of IL-8 and IL-10 were elevated in EC patients.
Zhang (81)	2014	Serum	EC: 64, HC: 26	ELISA	IL-10	Levels of IL-10 were elevated in EC patients.
Brooks (44)	2012	Serum	EC: 12, NC: 10	ELISA	IL-10, IL-17	No significant difference was found.
Cheng (82)	2020	Tissue	EC: 14, EH: 36	IHC	IL-17	IL-17A expression was increased in the EC.
Liao (83)	2020	Tissue	EC: 10, NED: 10	IHC	IL-24	IL-24 was overexpressed in EC samples.
Wang (26)	2021	Tissue	—	RNA-sequence	IL-32	IL-32 was overexpressed in EC and associated with longer overall survival time.

EH, endometrial hyperplasia.

Another member of the IL-10 family, IL-24, is also named MDA-7, which takes part in varieties of physiological activities under normal condition and has been emerged as an anti-tumor factor in multiple cancers. As reported by Liao et al. (83), the expression levels of IL-24 in EC patients were significantly increased when compared with that in health controls (**Table 3**). The expression levels of the rest 7 members of IL-10 family have not been reported till now and possess great research potential

### The other interleukins

IL-8, also known as CXCL-8, is a member of the CXC chemokine family that signals through two G protein-coupled receptors CXCR1 and CXCR2 (16). IL-8 produced in human endometrium is likely to intervene in the recruitment of neutrophils and lymphocytes into the endometrium (86). In EC cells, IL-1β (87), TNF-α (87), ulipristal acetate (68) and estrogen (88) can induce IL-8 expression, while polychlorinated biphenyls inhibited IL-8 expression through ER and AHR receptors (89). Additionally, it was covered that IL-8 expression was induced by prokineticin 1 in EC cell *via* activating the calcineurin/NFAT signaling which was negatively regulated by RCAN1-4 (90). As reported, IL-8 levels were dramatically elevated in patients with EC (23, 49, 79, 80) (**Table 3**). And the plasma levels of IL-8 in patients with EC were positively linearly correlated with visceral fat, which is a risk factor for EC (80). This hinted that IL-8 may be a bond linking the obesity and EC. Moreover, EC-derived CD133^+^ cells expressed more IL-8 than CD133^+^ hematopoietic progenitors did (91). Similarly, compared with normal fibroblasts, EC-associated fibroblasts secreted more IL-8 (60).

The IL-17 cytokine family contains 6 structurally related cytokines, including IL-17A, IL-17B, IL-17C, IL-17D, IL-17E (also called IL-25) and IL-17F, among which IL-17A is the prototypical member and is commonly known as IL-17 (18). IL-17 has been reported to participate in the oncogenesis and development of a broad spectrum of tumors. As for the expression of IL-17 in EC tissue (**Table 3**), studies (49, 82) showed that the mRNA and protein levels of IL-17 was both higher in EC lesions than normal endometrium. For the IL-17 levels in serum, Brooks et al. (44) reported no significant difference between EC patients and women without cancer, but this study has an obvious limitation of sample size that it only included 12 EC patients and 10 controls. By the way, it is worth noting that IL-17 signaling pathway was upregulated and enriched in EC supported by recent studies (92–95).

Besides, the serum level of IL-3 showed no difference between EC patients and healthy controls (96). Metformin treatment upregulated IL-5 expression in EC cells but downregulated IL-17A expression (39). IL-32 was overexpressed in EC compared with controls (26). There has been no report about IL-14, IL-16, IL-25, IL-34, IL-40 or the other interleukins in EC.

## Roles of interleukins in EC

**Figure 2:**
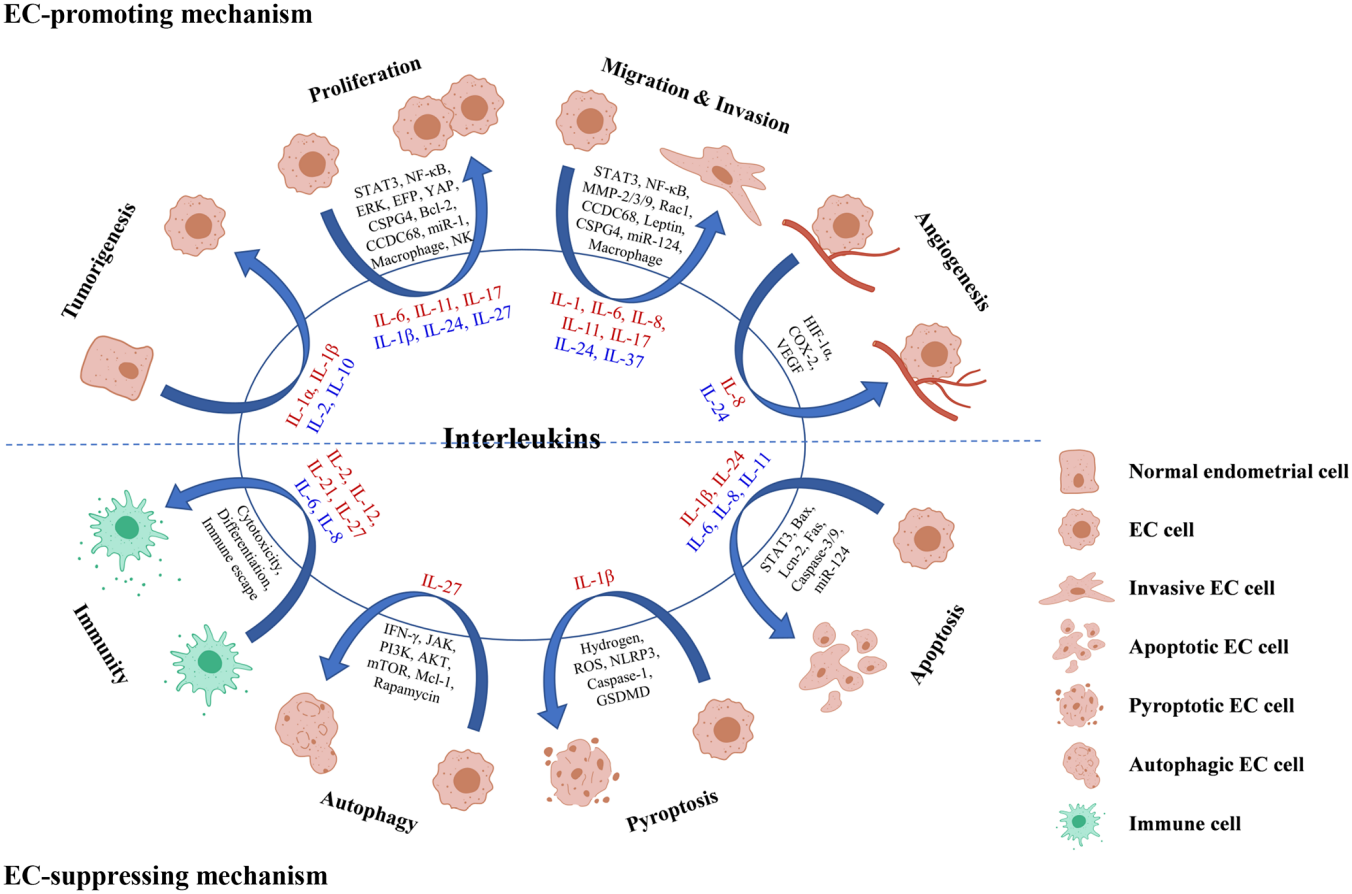
Roles and mechanisms of interleukins in EC oncogenesis and progression. Interleukin in red color means that it exerts a promoting effect on the process, while interleukin in blue color means that it exerts a suppressing effect on the process.

Increasing evidence has supported that interleukins played vital roles in the occurrence and development of EC *via* multiple pathways (**Figure 2**).

### Participating in tumorigenesis

The development of normal endometrium to EC is a long-term process from endometrial hyperplasia to atypia and finally to cancer. It was reported that IL-1α, IL-1β, IL-2 and IL-10 all play parts in the tumorigenesis of EC. Drugs including toremifene, genistin, daidzin, glycyrrhizae radix, glycyrrhizin, Juzen-taiho-to and Shimotsu-to inhibited the estrogen-related EC carcinogenesis by downregulating IL-1α (32–36). And the adverse histopathological changes in endometrium induced by estradiol benzoate can be reduced or prevented by diacerein *via* downregulating IL-1β (97), irbesartan *via* upregulating IL-10 (98) and melatonin *via* upregulating IL-2 (99).

### Regulating cell proliferation and growth

Growing evidence (100–104) have confirmed that IL-6 induced EC cells proliferation both *in vivo* and *in vitro*, and STAT3 played a vital part in this process. For instance, the application of IL-6 antibody reduced the tumor size stimulated by 17β-estradiol and decreased the expression of phosphorylated (p)- Stat3 in nucleus of EC cells (100). And IL-6 secreted by EC-associated fibroblasts (102) or adipose-derived stem cells treated with EC cells conditioned medium (104), promoted EC cell proliferation through STAT3 signaling. Additionally, IL-6-mediated EC proliferation can be inhibited by ERK/NF-κB pathway blockade (65) or CCDC68 knockdown (105); YAP-suppressed proliferation of EC cells can be attenuated by IL-6 (66); and estrogen-responsive finger protein (EFP) silencing can dampen the growth of EC by suppressing IL-6 cytokine family signal transducer, IL-10R1 and IL-26 (106).

IL-11 and IL-17 usually act as pro-oncogenic cytokines by facilitating malignant transformation and cancer cell progression. Nevertheless, there is still no consensus on the roles of them in EC. The earliest study by Lay et al. (107) declared that IL-11 had no effect on proliferation or viability of EC cells. But recent studies all supported the proliferation-promoting effect of IL-11 on EC cells with the upstream factors miR-1 (108) and YAP (66) as well as the downstream factor CSPG4 (109). With regarding to IL-17, Lai et al. (110) showed that IL-17 had little effect on HEC-1B cells growth. Ning et al. (111) suggested that IL-17A may not promote the cell growth directly but could have a vital effect on HEC-1A cells proliferation mediated by CD68^+^CD163^+^ macrophages. However, the latest study by Cheng et al. (82) confirmed the promoting effect of IL-17A on Ishikawa cells proliferation. The discrepancy among these studies may attribute to the differentiation grade and hormone receptor expression of cancer cells.

There are also interleukins which play proliferation-suppressing roles. IL-1ß had no effect on the proliferation of HHUA cells when used alone, yet a low concentration of which can enhance the inhibition effect of growth induced by Fas (112). The combination treatment of recombinant IL-2 (rIL-2) with lymphokine-activated killer (LAK) cells and lentinan drastically suppressed the growth of the EC in mice (113). The inhibition effect of IL-24 on Ishikawa cell proliferation and tumor growth was verified both *in vivo* and *in vitro*, which may be mediated by Bcl-2 downregulation (83). Moreover, IL-27 could not only directly inhibit the proliferation of EC cells but could also suppress the growth of EC cells by increasing the cytotoxicity of NK cells (20). Due to the inhibiting effect of IL-2, IL-24 and IL-27 on EC cells, there are bright prospects for them to be used as anti-tumor drugs.

### Modulating cell invasion and migration

STAT3 is deemed as a pivotal regulator of tumor metastasis because its target genes are implicated in multiple steps of tumor metastasis including cell invasion, migration. The effect of IL-6 and IL-11 on EC cell invasion and migration mainly mediated by the activation of STAT3 signaling (58, 103, 104, 107, 114, 115). For example, IL-6 secreted from adipose-derived stem cells promoted EC metastasis by activating STAT3 (104). Administration with miR-124 in EC cells can downregulate the expression of STAT3 by suppressing IL-6 and restrained cell invasion (103). Similarly, IL-11 promoted adhesion and migration *via* increasing p-Stat3 in EC cells (58, 107).

Matrix metalloproteinases (MMPs) has a history implicated in cancer for more than 50 years and is famous for its function of extracellular matrix degradation leading to cancer cell invasion and migration. As mentioned above, the upregulation of STAT3 by IL-6 was induced by MMP-2 (114). Recently, Che et al. (100) reported that IL-6 participated in E2-triggered migration and invasion of EC cells with the upregulation of MMP-2 as well. In addition to MMP-2, IL-6 can also increase the invasiveness of EC cells by inducing the release of MMP-9 (70, 116). Moreover, MMPs take part in the invasiveness of EC enhanced by IL-1α (31). While as tumor-suppressors, IL-24 dampened EC cell invasion by suppressing the expression of MMP-3 (83), and IL-37B inhibited the migration and invasion of EC cells *via* Rac1/NF-κB/MMP-2 signaling (30).

Apart from the above interleukins, IL-1, IL-8 and IL-17 also contributed a lot. As reported, the suppression of IL-1 signaling inhibited leptin-induced invasion of EC cells (4). For IL-8, it was detected that the mRNA and protein levels of IL-8 were higher in the metastatic variants of EC cells than that in the parent cell lines, which hinted a relationship between IL-8 and EC metastasis. Additionally, a significant correlation between infiltrated macrophage counts and IL-8 levels was noted in EC (117), and IL-8 secreted from infiltrated macrophages in EC is dramatically upregulated with myometrial invasion (118). And it was confirmed that IL-8 (119) and IL-8 signaling pathways (120) were of vital importance to EC metastasis. Besides, IL-17A likewise promoted the migration of EC cells (82).

Epithelial-mesenchymal transition (EMT) is a common trait of cancer invasiveness, which presents the acquirement of a mesenchymal morphology and decrease of epithelial markers. The expression of Snail, one of the mesenchymal markers, was increased by IL-6 (70) and IL-11 (109) *via* CSPG4 in EC cells, while another mesenchymal marker vimentin was downregulated by IL-11 blocking in EC cells (115). These results indicated that IL-6 and IL-11 could both enhance the EMT of EC. And verified by Winship et al. (115), the EMT-promoting effect of IL-11 was independent of IL-6. By the way, the migration and invasion induced by IL-6 in EC can be dramatically inhibited by CCDC68 (105).

### Modulating angiogenesis

Angiogenesis, the formation of new blood vessels from preexisting capillaries, is one of the essential steps in the development of solid tumors. HIF-1α, COX-2 and VEGF are all well-known promoters or mediators of cancer angiogenesis (121, 122). It was reported in EC that IL-8 expression was significantly correlated with microvessel counts (117); HIF-1α protein and mRNA levels were correlated with IL-8 expression (122); and COX-2 inhibitor declined IL-8 expression (121). All these indicated that IL-8 might act as an angiogenic switch in EC. Yet, there has been no direct evidence till now and further studies are needed. Contrary to IL-8, IL-24 is deemed as an inhibitor of angiogenesis *via* VEGF suppression (83). The overexpression of IL-24 was found to inhibit VEGF in Ishikawa cells and xenograft tumor. Meanwhile, fewer tumor blood vessels were observed in IL-24-Ishikawa group than in the Null-Ishikawa group.

### Regulating apoptosis

Apoptosis is an autonomous and orderly type of programmed cell death which possesses the mitochondrial endoplasmic reticulum-related intrinsic signaling pathway and death receptor-mediated exogenous signaling pathway. As for the former pathway, IL-24 appears to play a crucial part (83). The overexpression of IL-24 activated Bax, which induced the pores on mitochondria. Subsequently, apoptosome was formed with the activation of Caspase 9 followed by Caspase 3, finally causing the DNA damage and apoptosis of EC cells. In addition to IL-24, IL-1ß, IL-6, IL-8 and IL-11 are also associated with apoptosis of EC cells. IL-1ß was reported that can enhance the Fas-mediated apoptosis in HHUA cells by boosting post-receptor apoptotic signals (112). Downregulation of IL-6/STAT3 by administration of miR-124 in EC cells induced cell apoptosis (103). IL-8 suppressed Lcn-2-induced apoptosis of RL95-2 cells (119). Besides, the administration of IL-11Rα antibody was verified to promote EC apoptosis both *in vivo* (108) and *in vitro* (115).

### Promoting cell pyroptosis

Pyroptosis is a novel inflammatory programmed cell death pathway, which is generally accompanied with overproduction of the proinflammatory cytokines IL-1β and IL-18 (123). In EC, Yang et al. (21) proposed a model in EC cells that hydrogen promoted pyroptosis in EC *via* a ROS/NLRP3/Caspase-1/GSDMD pathway with the participation of IL-1β. Initially, hydrogen triggers the activation of ROS/NLRP3 signaling and subsequently the Caspase-1. With the activated Caspase-1, GSDMD is cleaved off the suppressor C-terminal domain and releases the pore-forming N-terminal domain, which then self-assembles to form pores in the plasma membrane. Finally, with IL-1β converting into its mature form, cellular pyroptosis is modulated. This study provided a scientific basis for developing the sensitizer to GSDMD-targeted therapy in the clinical management of EC.

### Improving cancer cell autophagy

Autophagy plays a two-sided role in cancer. On the one side, autophagy promotes cell survival in response to starvation or other cell stresses. On the other side, excessive autophagy can lead to the death of cancer cells. It was announced that IL-27 can inhibit IFN-γ-induced autophagy by concomitant induction of JAK/PI3K/AKT/mTOR cascade and up-regulation of Mcl-1 in macrophage (124). In EC, IL-27 was proved to enhance the rapamycin-mediated autophagy activation in tumor lesions and cells from Ishikawa-xenografted nude mice, although the induction of autophagy by rapamycin is not necessarily dependent on IL-27 (20).

### Regulating tumor immunity

Cytotoxicity of immunocyte is an important part of anti-tumor immunity. Interleukins including IL-2, IL-12, IL-21and IL-27 were found to be involved in increasing the cytotoxicity of immune cells to EC cells. IL-2 is a noted anti-tumor factor, low dose of which presented the ability of enhancing the antibody-dependent cellular cytotoxicity (ADCC) effect (125–128), as well as the cytotoxic effect mediated by immuno-conjugate molecule (hI-con1) (129) to USPC cells. Likewise, it was observed that IL-12, IL-21 and IL-12 plus IL-21 can all enhance the cytotoxicity of PBMCs isolated from EC patients versus control group, and the group of IL-21 plus IL-12 showed the most potent effect (130). This synergistic effect may be explained that IL-12 can enhance the production of IL-21 and IFN-γ in CD4^+^ T cells (131). Moreover, IL-27 increased cytotoxicity of NK cells through the upregulation of CD16, NKG2D, NKp46, perforin and Granzyme B together with the downregulation of KIR3DL1 and KIR2DL1 (20).

In addition to the effect on cytotoxicity, interleukins can affect the differentiation of lymphocyte as well. Based on the experiment *in vivo*, the suppression of IL-12 by miR-155 intervened with development of Th1 cell in the EC microenvironment (132). And *in vitro*, IL-12 plus IL-21 dramatically declined the rate of PBMCs apoptosis and the percentages of Treg cells but have no significant effect on the differentiation of Thl7 cells (130).

Unlike the anti-tumor effect of the above interleukins, IL-6 exerts a pro-tumor effect by promoting the immune escape of EC cells (133). It was observed in EC cells that IL-6 can cause the leakage of mitochondrial DNA *via* elevating the levels of NADPH oxidase and ROS. Then the downstream cGAS-STING signaling was activated in company with the increase of extracellular vesicle, which ultimately facilitated the immune escape of EC cells. And this effect can be eliminated by anti-PD-L1 treatment. Similarlly to IL-6, IL-8 also inhibits the immune function. It was covered that EC-derived mesenchymal stem cells secreted IL-8, which could decrease the proliferation of PBMCs (134).

Besides, it was mentioned that the levels of IL-2 were significantly elevated in Jurkat and T cells co-cultured with Ishikawa cells, which can be abolished by anti-PD-L1 antibody (135). CD39^+^CD103^+^ tumor-resident memory T cells sorted from human high-grade EC differentially expressed IL-2, IL-10, IL-13, IL-17A, IL-21, and IL-22 before and after activation (136). And IL-4 promoted the expression of HLA class II antigen DR and induced the expression of secretory component when estrogen was administrated (137).

## Clinical application of interleukins in EC

**Figure 3:**
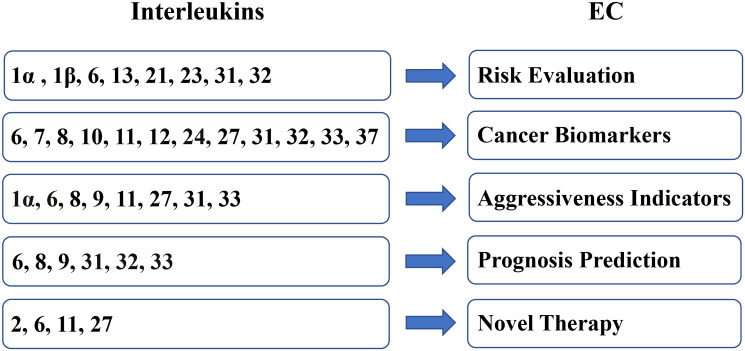
Clinical applications of interleukins in EC.

Owing to the expression difference between EC samples and benign samples and the tumor-regulating effect on EC, interleukins present a broad prospect for clinical applications in EC (**Figure 3**).

### Evaluating the tumorigenesis risk

Recent years with the development of genetic technology, the role of genetic factor in carcinogenesis has come into sight. The single nucleotide polymorphism (SNP) is the most common form of human genetic variations, which are significantly linked to the risk of tumorigenesis. It was reported that the polymorphisms in rs3783553, rs3783550, rs3783546, rs1609682 and rs3783521 of IL-1α (138, 139), rs1524107 and rs2066992 of IL-6 (140), rs4758680 and rs7977932 of IL-31 (141), rs28372698 and rs12934561 of IL-32 (2) may be relevant to increased susceptibility to EC in Chinese women. Moreover, the CC genotype of IL-6 Gene (−174 & -572) might contribute to EC risk (142).

Besides, there are multiple studies about the relationship between EC risk and interleukins. A case-cohort study (143) presented no relationship with EC risk in age- and/or BMI-adjusted models, while a case-control study (52) covered that the level of IL-6 was significantly associated with EC risk after adjusting age alone. Interestingly, among the patients with BMI < 25 kg/m^2^, IL-6 levels were significantly associated with an increased risk of EC (52). Taken together, IL-6 may be a risk indicator of EC, but the association is predominately dependent on the age or adiposity of patients. In addition, it was also mentioned that the serum levels of IL-1β, IL-13, IL-21 and IL-23 were negatively correlated with risk of EC (144).

### Acting as biomarkers

On the basis of literature, the levels of IL-6 (49–54), IL-8 (23, 49, 79, 80), IL-10 (23, 81), IL-31 (27, 29) and IL-33 (27–29) were all elevated in the serum and tissues of EC patients compared with healthy controls or patients with benign gynecological disorders. Additionally, the levels of IL-7 in serum (23) and IL-11 (57, 58), IL-24 (83), IL-32 (26) and IL-37 (30) in tissues as well as IL-11 in uterine flushing (58) were higher in EC patients. While the low levels of IL-12p70 (44) or IL-27 (20) may indicate the exist of EC. Besides, the mutation of IL-24 p.G192W existed only in EC tissues but not in non-cancerous tissues (145). The results above manifested that these interleukins are prospective biomarkers for EC diagnosis. However, more studies are needed to evaluate their diagnostic sensitivity and specificity as well as predictive values until they can be used as diagnostic parameters.

### Indicating the aggressiveness

In the light of studies, the levels of interleukin also had connections with the aggressive or progressive characteristics of EC. IL-6 and IL-8 were interleukins differentially expressed in type II EC and type I EC. Higher levels of IL-6 were detected in cell lines derived from USPC than cell lines of EAC, accompanied by the higher levels of IL-6 in patients with USPC (55). Similarly, SPEC-2 cells, established from stage IV USPC, expressed more IL-8 mRNA than HEC-1A cells from stage IA EAC (87). The differentiation grade of EC is associated with the expression of interleukins too. Elevated levels of IL-1α (22), IL-11 (57), IL-31 (27) and IL-33 (27) were observed in poor differentiated EC, while low level of IL-27 (20) and infiltration of IL-9^+^ lymphocyte (47) was recorded with the decrease of EC differentiation degree. Moreover, IL-6 (56, 146), IL-8 (117), IL-31 (27, 29) and IL-33 (27, 29) were positively correlated with poor clinical characteristics including advanced stage, myoinvasion, and node or distant metastases. Therefore, in the future, these interleukins may be useful as the indicators for the management of EC, for example for the evaluation of whether lymph node dissection is necessary.

### Predicting the prognosis

Some interleukins also exhibit value in the aspect of prognosis predicting in EC. According to the literature, IL-6 (147) and IL-8 (79), as the upregulated and tumor-promoting interleukins, were significantly linked to low overall survival in patients with EC. Similarly, the IL-31/IL-31R and IL-33/ST2 system can be indicators due to the correlation between strong expression of IL-31R or ST2 and poor survival of EC patients (27). On the contrary, the infiltration with IL-9^+^ lymphocyte (47) or increased level of IL-32 (26) was identified as better prognostic factors of EC.

### Serving as the immune therapy

It was proclaimed that the killer cells activated by IL-2 have a capacity to distinguish normal endometrial cells from malignant endometrial cells (148), which made it prospective for IL-2 acting as an anti-tumor cytokine. Studies in earlier years reported that the growth of the EC in mice was considerably suppressed by the combination therapy of LAK cells, rIL-2 and lentinan (113), and EC patients had no response to the therapy of LAK cells plus rIL-2 (149) while had partial responses to the therapy of rIL-2 plus rIFN-α (150). Later, scholars noted that low dose of IL-2 can enhance the cytotoxicity of immunocyte and the efficacy of anti-tumor drugs, including hRS7, hI-con1, herceptin, adecatumumab, trastuzumab and pertuzumab (125–129) in USPC patients. In addition to IL-2, IL-27 was recognized as another therapeutic interleukin against EC. Rapamycin was found to simultaneously upregulate the expression of IL-27 in EC cells and IL-27R on NK cells, which further increased the cytotoxic activity of NK cells to EC cells and autophagy activation of EC cells (20). Moreover, the combination of rapamycin and cisplatin exerts a better anti-EC effect than rapamycin or cisplatin alone by the IL-27-mediated cytotoxicity activation of NK cells. These results suggested that IL-2 and IL-27 may be useful assistant reagents for anti-EC therapy.

STAT3 signaling pathway, as a pivotal player in EC pathogenesis, could potentially be employed as the target of treatment. As reported, inhibition of the downstream effectors JAK1 and STAT3 of IL-6 dampened the growth of EC both *in vitro* and *in vivo* (101). The combination of IL-6 signaling inhibitors with cisplatin can increase the sensitivity of cisplatin-resistant ALDH^hi^ EC cells (101). As well, anti-IL-6R antibody treatment was proved to be useful in shrinking the EC tumor (51, 101). Besides, the blockade of IL-11 signaling with IL-11Rα antibody caused the reduction of EC cell viability and proliferation and impairment to cell metastasis *in vitro*, together with the inhibition of tumor growth and induction of cell apoptosis in EC xenograft models (108, 115). In summary, IL-6 and IL-11 might be useful therapeutic targets in EC, but further preclinical experiments and clinical trials are needed.

## Conclusion

Present literature has confirmed that interleukins play crucial roles in the oncogenesis and development of EC *via* regulating the proliferation, migration, invasion, angiogenesis, apoptosis, pyroptosis and autophagy of EC as well as the immune function against EC. And some of the interleukins seems to have prospective clinical applications in EC, such as evaluating the risk of tumorigenesis, discriminating the malignancy from benign disorders or normal condition, indicating cancer aggressiveness, predicting the prognosis of patients and serving as immune therapy.

However, there are still some limitations in the present studies. First of all, the discrepancy among results of different studies can not be ignored, which may be improved by enlarging the sample size, normalizing the inclusion standard and using high-sensitivity detection methods. In addition to the traditional detection methods including ELISA, IHC and PCR, more advanced methods can also be used such as Luminex Assay, Meso Scale Discovery, Multiplex immunofluorescence and RNA-sequence, which can detect multiple cytokines simultaneously with adequate sensitivity and accuracy. What’s more, all the published studies have not taken the molecular classifications of EC by TCGA into consideration, which would lead to a deficiency in the relationship between interleukins and genomic features of EC. So in the future, it would be better for studies to include an analysis of interleukins and these molecular sub-groups. And last but not least, the effect of interleukins on EC and the mechanism have not been elucidated completely and more experiments both *in vitro* and *in vivo* are needed. Therefore, there is still a long way to go before the clinical applications of interleukins in EC come into reality. Nevertheless, it is certain that the exploration of interleukins will definitely be of great benefit to the screening, diagnosis and treatment of EC in the future.

## Author contributions

YuqinZ conceived the framework, searched literature, integrated data and wrote the manuscript. HL searched literature, integrated data and wrote the manuscript. SL and RZ reviewed the manuscript. KZ and YuqiZ helped with the tables and figures. YW and FX reviewed the paper and provided fundings. All authors contributed to the article and approved the submitted version.

## Funding

This work was supported by National Natural Science Foundation of China (81972448) and Tianjin Science and Technology Project, China (20JCZDJC00330)

## Conflict of interest

The authors declare that the research was conducted in the absence of any commercial or financial relationships that could be construed as a potential conflict of interest.

## Publisher’s note

All claims expressed in this article are solely those of the authors and do not necessarily represent those of their affiliated organizations, or those of the publisher, the editors and the reviewers. Any product that may be evaluated in this article, or claim that may be made by its manufacturer, is not guaranteed or endorsed by the publisher.
